# Decomposing socioeconomic inequality in blood pressure and blood glucose testing: evidence from four districts in Kerala, India

**DOI:** 10.1186/s12939-022-01737-x

**Published:** 2022-09-09

**Authors:** Santosh Kumar Sharma, Devaki Nambiar, Hari Sankar, Jaison Joseph, Surya Surendran, Gloria Benny

**Affiliations:** 1 The George Institute for Global Health, New Delhi, India; 2 Health Systems and Equity, The George Institute for Global Health, New Delhi, India; 3 Faculty of Medicine, University of New South Wales, Sydney, Australia; 4 Prasanna School of Public Health, Manipal Academy of Higher Education, Manipal, India

## Abstract

**Background:**

Non-Communicable Diseases (NCDs) constitute a significant danger to the nation’s public health system, both in terms of morbidity and mortality, as well as the financial burden they inflict. Kerala is undergoing an epidemiologic transition, which has significantly impacted the state’s morbidity and mortality figures. For decades, the state has been putting in place myriad programs to reduce the burden of NCDs across population groups. Socioeconomic inequalities in NCD testing have been documented in India, although they are understudied in Kerala. The study aimed to estimate and characterize districtwise socioeconomic inequality in Blood Pressure (BP) and Blood Glucose (BG) testing.

**Methods:**

A cross-sectional household survey was conducted between July–October 2019 in Kasaragod, Alappuzha, Kollam and Thiruvananthapuram districts of Kerala, India. A total of 6383 participants aged 30 years and above were interviewed using multistage random sampling. Descriptive statistics were derived district-wise. We computed ratios, differences, equiplots, and Erreygers concentration indices for each district to measure socioeconomic inequality in BP and BG testing. Erreygers decomposition techniques were used to estimate the relative contribution of covariates to socioeconomic inequality.

**Results:**

There was a significant concentration of BP and BG testing favouring wealthier quintiles in Alappuzha, Kollam, and Thiruvananthapuram districts. The inequality in BP and BG testing was highest in Thiruvananthapuram (0.087 and 0.110), followed by Kollam (0.077 and 0.090), Alappuzha (0.083 and 0.073) and Kasaragod (0.026 and 0.056). Decomposition analysis revealed that wealth quintile and education contributed substantially to socioeconomic inequality in BP and BG testing in all four districts. It was also found that family history of NCDs significantly contributed to observed socioeconomic inequality in BP testing (29, 11, 16, and 27% in Kasaragod, Alappuzha, Kollam, and Thiruvananthapuram, respectively). Similarly, in BG testing, family history of NCDs substantially contributed to observed socioeconomic inequality, explaining 16–17% in Kasaragod, Alappuzha, Kollam, and Thiruvananthapuram respectively of the total inequality.

**Conclusion:**

While the magnitude of socioeconomic inequality in NCD risk factor testing did not appear to be very high in four Kerala districts, although levels were statistically significant in three of them. Greater exploration is needed on how education and caste contribute to these inequalities and their relationship to NCD risk factors such as family history. From such analyses, we may be able to identify entry points to mitigate inequalities in testing access, as well as burden.

**Supplementary Information:**

The online version contains supplementary material available at 10.1186/s12939-022-01737-x.

## Background

Non-Communicable Diseases (NCDs) are becoming more prevalent throughout the world and account for a greater share of disability-adjusted life-years than communicable diseases over the past two decades or so [1, 2]. Addressing NCDs will be pivotal to achieving various Sustainable Development Goals (SDGs) and by 2030 may reduce premature deaths by about a third [3, 4]. While Low- and Middle-Income Countries (LMICs) account for nearly half of all premature NCD deaths, the problem of NCDs is steadily worsening due to socioeconomic disparities [5]. Cardio-Vascular Disease (CVD), cancers, diabetes, and respiratory disorders, such as asthma and Chronic Obstructive Pulmonary Disease (COPD), are the leading causes of NCD fatalities [6]. NCDs have long been seen to be an issue primarily affecting High-Income Countries (HICs); nevertheless, the proportion of morbidity caused by NCDs in LMICs is rising [7, 8]. NCDs and injuries account for more than two-thirds (67%) of India’s disease burden, as assessed by disability-adjusted life years (DALYs) [9]. The disease burden estimates at the state level in India reveal considerable variations in disease burden composition between states [9]. Kerala is at a relatively advanced stage of both the epidemiological and demographic transitions as compared to other states of India and thereby contends with a substantial NCD burden [10, 11].

The government of India launched the National Programme for Prevention and Control of Cancer, Diabetes, Cardiovascular Diseases and Stroke (NPCDCS) in 2010 with an emphasis on infrastructure, human resource development, health promotion, early diagnosis, management, and referral (NHM & MOHFW, 2022). Under the program, NCD Cells were established at National, State and District levels to provide free diagnostic facilities and drugs for patients. As on March 2016, the program has been implemented in all the 36 States/UTs with 298 districts NCDS Cells and 293 NCD clinics [12]. Kerala’s Health Services Department has launched an initiative known as ‘Amrutham Aarogyam,’ in 2017, a state-wide initiative which has reached out to the public and assisted in the identification of over ten lakh diabetic and hypertension patients [13]. Amrutham Arogyam includes services provided by all district/general hospitals, sub-district level hospitals, Community Health Centers, Primary Health Centers, and even the 5400 sub-centers that serve a population of 5000 people. Kerala prides in being the only state with a comprehensive NCD screening programme that spans the whole health system [13].

Inequalities in health have been seen all across the world, regardless of the measure of socioeconomic position applied [14, 15, 16, 17]. At both the national and subnational levels, these inequalities have been shown to be widespread. As a result, a key policy problem is determining the effects of social, economic, and demographic factors on health. In several countries, the burden of most NCD risk factors is shown to be higher among impoverished and marginalised persons and groups than among those with better Socioeconomic Status (SES) [16, 17, 18, 19, 20]. In LMICs, studies on the relationship between socioeconomic status and NCDs are uncommon, and there is scant systematic evidence to support this relationship between socioeconomic position and health [20].

A number of studies argue that a health system delivers universal health coverage when everyone, regardless of socioeconomic background, receives necessary, high-quality health treatments without undue financial burden [21, 22]. Contrary to popular belief, socioeconomic disparities and discrepancies in the use of NCD care services occur even in high-income nations with universal healthcare [23, 24, 25]. In addition, several studies conducted in LMICs have documented socioeconomic inequalities in the awareness, treatment, and control of hypertension found a pro-rich inequality in the levels of hypertension awareness and treatment [26, 27, 28, 29].

Evidence suggests that factors such as socioeconomic status, area of residence, and to an extent, sex, education status, age and having health insurance explain socioeconomic inequalities in care utilisation for NCDs in LMICs [2, 26, 29, 30, 31].

Socioeconomic disparities in the diagnosis and treatment of NCDs, particularly BP and BG, have been explored in several studies [22, 32, 33, 34, 35, 36]. These studies mostly concentrated on national and state-level estimates, ignoring disparities at the subnational and district levels, which are more important to policymakers’ and administrators’ needs. Therefore, this study attempts to address the gap in research and examining the district level in(equity) in the testing of BP and BG in Kerala. Also using decomposition analysis, we explored socioeconomic factors associated with inequality in the testing of BP and BG in the selected districts of Kerala.

## Data and methods

### Study design and setting

**Fig. 1:**
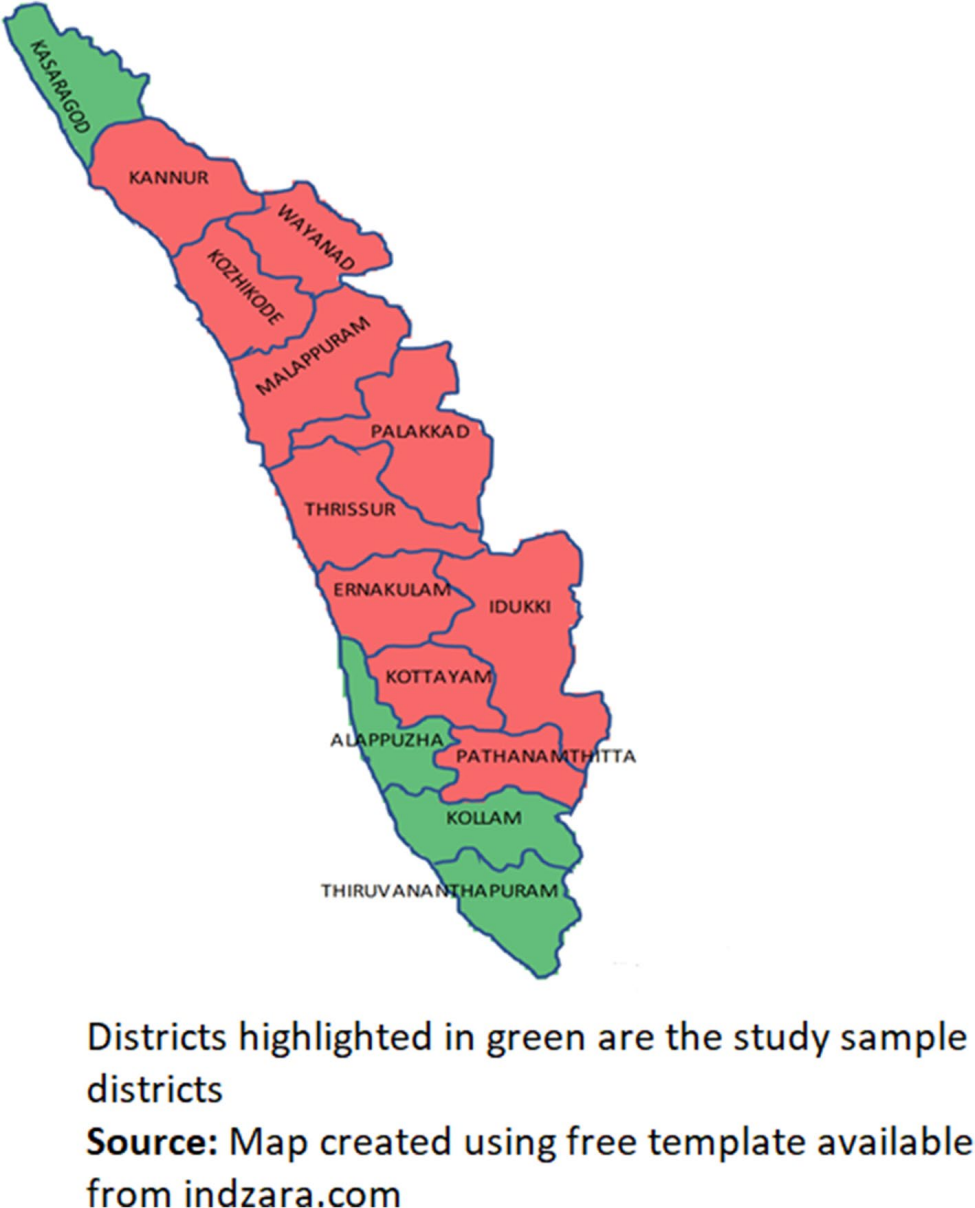
Selected Districts in Kerala

We used a cross-sectional analysis of a household survey conducted during July–October 2019 in four districts of Kerala. Principal Component Analysis (PCA) a dimension-reduction tool that helps in concentrating the information of a large data to a smaller one was used to classify the fourteen districts of the state in four groups (Fig. 1). The selected health indicators and data on determinants of health sourced variables from NFHS [37] data used for district classification were i) household with electricity, ii)improved drinking water, iii) improved sanitation, iv) clean fuel for cooking, insurance v) Women with 10 or more years of schooling (%), vi)high glucose in women and men, vii) women who ever had cervix examination, viii) women with below normal Body Mass Index (BMI), ix)above normal BMI, x) children with full immunization, xi) hypertension in men and women, and xii)children reporting Diarrhoea in last 2 weeks. One district was randomly selected from each of four groups of districts using random.org an online open-source randomizer tool. The list of Family Health Centres (FHC) and Primary Health Centre (PHC) in that selected district at that time period (June2019) was obtained from department of health Kerala. From the list one FHC and one PHC were randomly selected. Multistage random sampling method was used to collect the information form the household and individuals. The data from NCD control programme of eligible males and females above age thirty who had checked the blood pressure intime period of 2018–19 and a design effect of 2 was used for calculating the required sample size sample for the study. The population served the FHC/PHC were the population of interest. The health facility catchment area (HFCA) also correspond to the grama panchayath area (administrative demarcation of villages by government of India). Each HFCA were constituted by wards which were considered as our Primary Sampling Unit (PSU). The wards of our study sites consisted of 300 households (data provided by department of health). Twenty wards were selected from each HFCA. In areas where there was less than or equal to 20 wards all were selected and HFCA with more than twenty wards were divided to five clusters and four wards from each clusters were randomly selected. The study team creates outline maps of wards with roads land marks and households with support from elected representatives of the ward, field health workers and. Each PSU with ward households more than 300 were divided to 2–3 segments in these maps and twenty demarcated artificial ward segments were created for each HCFA. Twenty household were randomly selected from each of these ward segments using systematic random sampling. Th inclusion criteria was decided as household which had at least one member above 30 years. The detailed sampling design, survey method, data collection, sample size calculation and data processing are reported elsewhere [32, 38].

### Study tool

A household survey using multistage random sampling design was conducted to collect information from 3234 households in the selected eight FHC/PHC catchment areas of four districts in Kerala.

The questionnaire designed for the study had multiple blocks on individual demographics and other health information, hospitalisation, outpatient care, NCDs, satisfaction with FHC/PHC visits, and awareness of services under the Aardram mission. This study used the data from NCDs blocks to gather information on selected indicators. The questionnaire is attached to the Supplementary file.

The proportion of eligible participants aged 30 & above whose Blood Pressure (BP) and Blood Glucose (BG) measured in the previous year was obtained from data provided by NCD program of the Kerala Health Department. One person aged 30 or older was randomly selected from each household to get information on NCD testing and risk factors high blood pressure and high blood glucose level (HBP and HBG). Out of total sample size of 13,064 individuals of all ages, the information about NCDs were only collected from 6383 individual aged 30 and above.

Ethics approval of the study was received from the institutional ethics committee of George Institute for Global Health (Project Number 05/2019). All participants provided written informed consent before taking part in the study.

## Variables of the study

### Outcome variable

The main outcome variables for the study were self-report of blood pressure and blood glucose testing by a medical professional in the previous year among respondents aged 30 and above. Participants were asked “When was the last time you or doctor/doctor/nurse/other medical personnel measured your blood pressure (BP)” (the same question was asked separately for blood glucose (BG)). They were considered to have been “tested” if they responded with “during last six months or last year”.

### Independent variable

A set of independent variables were used in the analysis. Selected independent variables included in the study are age group (30–44, 45–59, 60+), sex (male, female), education (illiterate, primary, secondary and higher secondary & above), religion (Hindu, Muslim, Christian, Others (Sikhs, Jain, etc.)), social caste group (Scheduled Caste [SC], Scheduled Tribe [ST], Other Backward Caste [OBC], Other), household wealth quintile (poorest, poorer, middle, richer, richest), family history of high blood pressure, high glucose level and heart diseases (no, yes). These variables were decided based on the variable availability in the study data set and well-established literature [20, 29, 31, 32, 39, 40, 41].

Drawing on some of the classification systems of the colonial era, the Indian government created a caste classification scheme in contemporary India, dividing the caste groups facing historical disadvantage into scheduled castes (SC), indigenous groups (some of whom were earlier criminalised) into scheduled tribes (ST), and other castes facing disadvantage as Other Backward Castes (OBC). The remaining population is considered to be a general caste. A household wealth index was used as a proxy for household economic status. The wealth index was created using economic proxies such as household durable assets, access to safe drinking water and sanitation, and landholding. The index was produced by dividing the data into sets of dichotomous variables and then using principal component analysis (PCA) to give indicator weights. The resulting wealth index was split into five quintiles: poorest, poorer, middle, richer, and richest [42]. Family history of NCDs was created by asking question to respondent as “Does any family member (parents or siblings) have high blood pressure, diabetes, or heart disease?”

## Methods

Descriptive statistics (mean, standard error (SE) and 95% confidence interval) was carried out for the district wise analysis. We have used inequality ratio and inequality gap measures of inequality. The inequality ratio is defined as the ratio between the proportion of respondents tested for BP and BG among the poorest to the proportion of respondents tested for BP and BG among the richest wealth quintiles. The inequality gap is defined as the absolute difference in the proportion of respondents tested for BP and BG between two extreme groups of wealth quintile. We also used equiplots to display inequalities in BP and BG testing by socioeconomic status using the equiplot.ado file.

Concentration curves and concentration indices were computed to discern inequality in BP testing and BG testing in the districts of Kerala.

### Choice of concentration index

Concentration curve plotted the cumulative proportions of health variable against the cumulative proportions of sample ranked by the equivalized household wealth index (assets index). The standard concentration index, denoted below by C, is computed as twice the area between the concentration curve and the line of equality (the 45-degree line) [43]. Formally, the CCI is defined as

1 $$\mathrm{C}=\kern0.75em \frac{2}{n\mu}\ \sum\nolimits_{i=1}^n hiRi-1$$

Where, *μ* is the mean of the health outcome variable *h*_*i*_; *R*_*i*_ is the rank of the individual in the wealth distribution. The magnitude of the CCI represents both the intensity of the association and the degree of variability in the health variable, and its value shows the direction of relationship between the health variable and position in the living standards distribution [44].

In this study, the outcome variables are binary characterized by ordinal or bounded nature,, therefore, they are not compatible with rank dependent measures such as concentration index measuring relative inequality as it does not allow for differences between individuals to be compared [45, 46]. With binary health outcome variables, the concentration index will not lie within normal bands but between *μ* − 1 and 1 − *μ* for large samples. This suggests the need for some form of normalization. Therefore, we used concentration index (CCI) with Erreygers’ correction, a quasi-absolute measures appropriate for binary health outcome [47, 48], can be written as

2 $$Ec=\left(4\mu \right)/\left(b-a\right).C$$

Where, C = standard concentration index as denoted in Eq. 1, *μ* is the mean of the health outcome variable with its range defined (*b* − *a*) (b is the upper bound, and a is the lower bound). A negative value of CCI suggests concentration of health outcome variable in lower levels of socioeconomic status, and a positive value indicates concentration among those who are more affluent.

## Decomposition of concentration index

Decomposition of Erreygers concentration index was conducted in order to determine the relative contribution of covariates in explaining the inequality and unexplained residuals. The decomposition of CCI was conducted using a probit regression models, which gives the estimation of a) covariate-specific concentration, b) elasticity, the direction and magnitude of the relationship between a variable and health, and c) a percentage contribution of a covariate to overall inequality, summarized by the CCI [49]. Covariate-specific concentration indices show distribution of a factor along the socioeconomic continuum. Elasticity informs about the probability of poor health. In the case of ratio-scale variables, a classic economic interpretation is possible (e.g., what percent change in chances of a disease is predicted by a 1% increase in a covariate). In the case of dummy variables, the sign of the elasticity informs about whether there is a health benefit or disadvantage associated with belonging to a category, in comparison to a reference category. A product of covariate-specific CI and its elasticity informs about what concentration of a disease is produced by a covariate alone, presented in absolute terms. Division of the absolute contribution by the overall magnitude of health inequality, as measured by Erreygers CCI, produces a percentage contribution of a covariate to overall health inequality. Results of the decomposition analysis are presented in tornado graphs, as per convention.

All the statistical analyses were conducted in Stata®17 (StataCorp LLC, Lakeway Drive College Station, Texas, USA), using appropriate survey weights.

## Results

Table 1 presents the percentage distribution of respondents who have given responses on NCDs according to socio-demographic and economic characteristics by selected districts in Kerala. As shown in table, a higher proportion of respondents were female (54% in Kasaragod & Kollam, 52% in Alappuzha, and 53% in Thiruvananthapuram) in the 45 & above years category in selected districts of Kerala. A majority of respondents had attained secondary and higher secondary level of education. In all the districts, the proportion of Hindus (88% in Kasaragod, 66% in Alappuzha, 48% in Kollam, and 68% in Thiruvananthapuram) were higher than other religious groups. Similarly, a majority of respondents belong to the Other Backward Caste (OBC), 77% in Kasaragod, 66% in Alappuzha, 61% in Kollam, and 57% in Thiruvananthapuram. About 64% respondents in Thiruvananthapuram had the family history of NCDs followed by Kasaragod (51%), Kollam (49%), and Alappuzha (48%).

**Table 1:** Percentage distribution of respondents who have given responses on non-communicable diseases according to socio-demographic and economic characteristics by selected districts of Kerala

Background characteristics	n	Kasaragod	n	Alappuzha	n	Kollam	n	Thiruvananthapuram	N	Total
**Age**										
30–44	403	22.3	418	24.4	457	27.4	438	27.6	1716	26.1
45–59	646	37.3	591	34.8	574	35.8	591	38.5	2402	36.8
60+	583	40.4	605	40.8	568	36.8	509	34.0	2265	37.1
**Sex**										
Male	731	45.7	775	48.4	733	45.6	723	47.3	2962	46.7
Female	901	54.3	838	51.7	865	54.4	813	52.7	3417	53.3
**Education**										
Illiterate	90	6.2	35	2.8	79	6.0	75	5.9	279	5.4
Primary	101	7.1	12	1.2	8	0.5	7	0.4	128	1.7
Secondary	694	43.2	605	39.4	514	34.0	471	29.9	2284	35.0
Higher secondary & above	746	43.5	962	56.6	998	59.5	985	63.7	3691	57.9
**Religion**										
Hindu	1482	88.2	1130	66.3	961	48.3	1050	68.0	4623	65.3
Non-Hindu	132	10.9	238	18.7	381	32.0	159	10.1	910	18.2
Christian	18	0.9	246	15.1	257	19.7	329	21.9	850	16.6
**Caste**										
SC/ST	73	5.6	88	5.7	174	7.5	209	14.5	544	9.4
OBC	1257	76.8	1069	65.8	861	60.6	898	56.8	4085	62.8
Others	302	17.6	457	28.5	564	31.9	431	28.8	1754	27.8
**Wealth quintile**										
Poorest	443	19.9	240	13.6	268	15.3	352	20.8	1303	17.7
Poorer	296	17.2	282	15.4	364	21.5	341	21.2	1283	19.6
Middle	297	19.0	355	22.1	342	19.7	312	20.8	1306	20.4
Richer	269	21.0	362	23.9	355	23.3	274	18.2	1260	21.2
Richest	327	22.9	375	25.0	270	20.3	259	19.0	1231	21.1
**Family history of HBP and HBG and heart diseases**										
No	794	48.9	839	52.4	808	51.4	569	36.0	3010	45.6
Yes	838	51.1	775	47.6	791	48.6	969	64.1	3361	54.4
Total (N)	1632	100.0	1614	100.0	1599	100.0	1538	100.0	6383	100.0

**Fig. 2:**
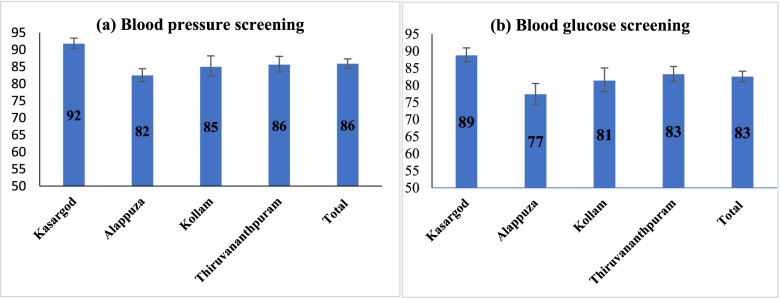
Percentage distribution of respondents whose blood pressure and blood glucose measured by doctor/nurse/other medical personnel in the previous year

Figure 2 and Table A1 presents the percentage distribution of respondents whose BP and BG were measured by doctor/nurse/other medical personnel in the last 12 months in the selected districts of Kerala. BP testing was highest in Kasaragod [92%; 95%CI: 90.11, 93.13), followed by Thiruvananthapuram [86, 95%CI: 83.18, 87.74], Kollam [85%; 95%CI: 81.76, 87.71], and Alappuzha [82, 95%CI: 80.50, 84.28]. Similarly, BG testing was also highest in Kasaragod, followed by Thiruvananthapuram, Kollam, and Alappuzha.

**Fig. 3:**
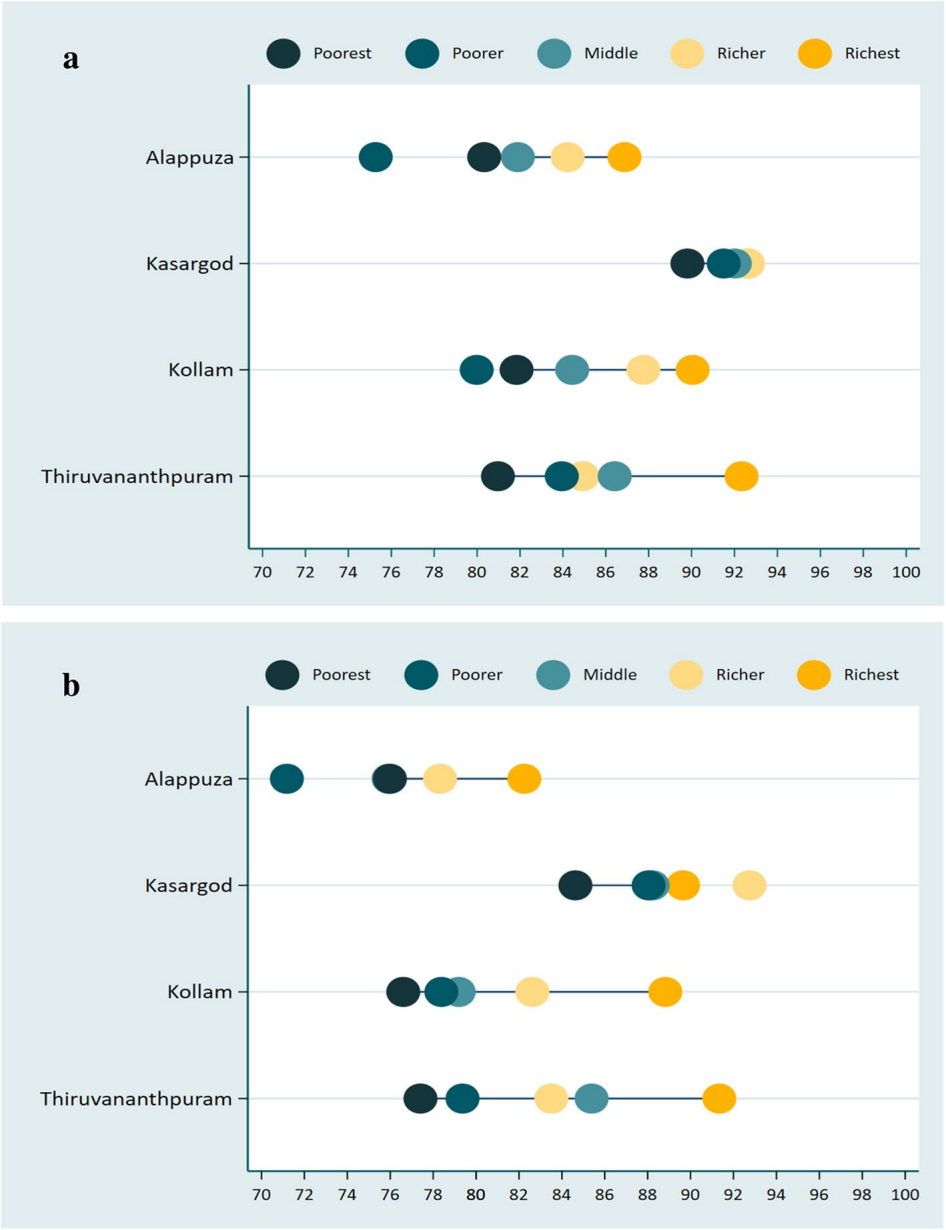
**a** Blood pressure testing by wealth quintile in the districts. **b** Blood glucose testing by wealth quintile in the districts

Figure 3a and b show equiplots of BP testing and BG testing coverage for four selected districts included in this study, by wealth quintile. The higher the coverage, the more to the right the series of dots is. From the lowest, or Q1 (black dot), to the richest, or Q5 (gold dot), each dot symbolises one wealth quintile. A line connects these two dots; longer lines reflect bigger absolute disparities. In most cases, the five quintiles are ordered from left to right, as coverage is lowest on Q1 and increases with wealth. In Alappuzha, Kollam and Thiruvananthapuram, the richest quintile had a substantially higher proportion of testing coverage than all other quintiles, that is, the richest quintiles are well ahead of the rest of the population, whereas in Kasaragod, there was a very little gap between richest and poorest quintile for the BP and BG testing. Thiruvananthapuram and Kollam districts presents the largest absolute wealth inequality in BP and BG testing in Kerala.

Table A2 presents the wealth inequality ratio (that is the relative difference in the proportion of respondents measured for blood pressure and blood glucose between extreme wealth quintile), and the inequality gap (i. e., the absolute difference in the proportion of respondents measured for blood pressure between extreme wealth quintile) in the districts of Kerala. The result of rich-poor ratio and rich-poor difference revealed that respondents from the richest households were more likely to get tested for blood pressure and blood glucose than the respondents from the poorest households in all the selected districts of Kerala. More particularly, the value of rich-poor ratio and rich-poor difference for the blood pressure testing was highest in Thiruvananthapuram (1.14 and 11.36), followed by Kollam (1.10 and 8.20), and Alappuzha (1.08 and 6.54). Similarly, the value of rich-poor ratio and rich-poor difference for the blood glucose testing was highest in Thiruvananthapuram (1.18 and 13.93), followed by Kollam (1.16 and 12.22), Alappuzha (1.08 and 6.27). This means that socio-economic inequality in blood pressure and blood glucose testing was higher among affluent class of household in the selected districts of Kerala.

Table 2 presents the concentration index (CCI) of BP testing and BG testing in the four selected districts of Kerala. Socioeconomic inequalities with respect to BP and BG testing refer to the degree to which BP and BG testing rates differs between less or more economically advantageous groups. The positive value of CCI clearly shows that BP testing and BG testing are concentrated more in affluent class of households. The value of CCI for BP testing shows that the inequality was significantly higher in Thiruvananthapuram (CCI = 0.087; SE = 0.021), Alappuzha (CCI = 0.083; SE = 0.022), and Kollam (CCI = 0.077; SE =0.021) than the Kasaragod district (CCI = 0.026; SE = 0.016). Similarly, the inequality in BG testing was highest in Thiruvananthapuram (CCI = 0.110; SE = 0.022), followed by Kollam (CCI = 0.090; SE = 0.022), Alappuzha (CCI: 0.073; SE = 0.024), and Kasaragod (CCI = 0.056, SE = 0.018).

**Table 2:** Concentration Index for blood pressure testing and blood glucose testing in Kerala

Districts	Blood pressure testing			Blood glucose testing		
	CCI	SE	*p* value	CCI	SE	*p* value
Kasaragod	0.026	0.016	0.097	0.056	0.018	0.002
Alappuzha	0.083	0.022	0.000	0.073	0.024	0.002
Kollam	0.077	0.021	0.000	0.090	0.022	0.000
Thiruvananthapuram	0.087	0.021	0.000	0.110	0.022	0.000
Total	0.069	0.010	0.000	0.083	0.011	0.000

**Fig. 4:**
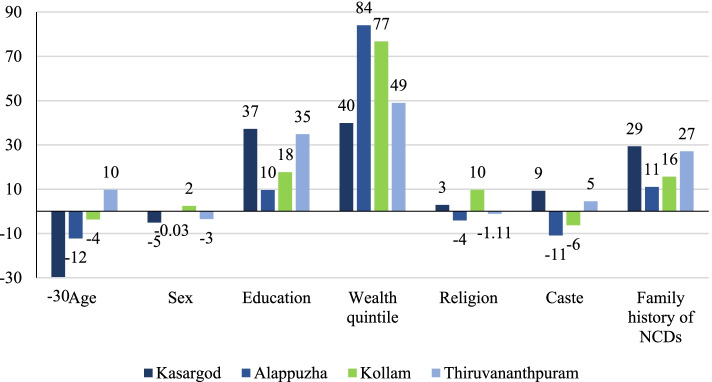
Decomposition of the CCI of blood pressure testing

Decomposition results show that the largest contribution to inequality in BP testing was attributable to wealth quintile in Alappuzha (84%), Kollam (77%), Thiruvananthapuram (49%) and Kasaragod (40%) (see Fig. 4, Appendix Tables A3, A4). Similarly, education made a substantial contribution to observed inequality in BP testing, explaining 37% in Kasaragod, 35% in Thiruvananthapuram, 18% in Kollam, and 10% in Alappuzha of the total inequality. It was found that family history of NCDs contributed significantly to observed inequality in BP testing in all the selected districts of Kerala, explaining 29% in Kasaragod, 11% in Alappuzha, 16% in Kollam, and 27% in Thiruvananthapuram. This finding could be said to be expected in a scenario where the overall magnitudes of inequality in other domains are relatively low; however, further inquiry is required. In some cases, since participants were responding on behalf of other family members making it possible that reporting errors are greater in relation to this variable compared to others. Iven the high levels of literacy in the state, however, and the typical role (especially financial) of families in care-seeking, we may reasonably assume reporting error to be relatively low, Should such biases be minimal, however, given the intergenerational nature of both socioeconomic inequality and risk for NCDs, this finding is not surprising. The positive value for wealth quintile, education, and family history of NCDs indicated that if BP testing were determined by these factors, it would be pro-rich. Negative contribution was most apparent for age (− 30% in Kasaragod, − 12% in Alappuzha, − 4% in Kollam), caste (− 11% in Alappuzha, − 6% in Kollam), religion (− 4% in Alappuzha) indicating concentration in favor of economically backword sections.

**Fig. 5:**
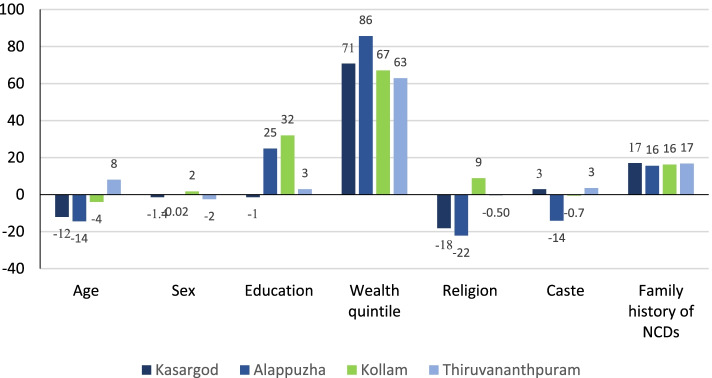
Decomposition of the CCI of blood glucose testing

Similarly, Fig. 5 and appendix Table A5 and A6 presents the decomposition results for BG testing among individuals aged 30 & above. The largest contribution to inequality in BG testing came from the wealth quintile in Alappuzha (86%), Kasaragod (71%), Kollam (67%), and Thiruvananthapuram (63%). Education showed a significant contribution to observed inequality in BG testing in Kollam (32%), Alappuzha (25%), and Thiruvananthapuram (3%). Similarly, family history of NCDs also showed a significant contribution to observed inequality in BG testing in the all the selected district included in the study, explaining 17, 16, 16 and 17% in Kasaragod, Alappuzha, Kollam, and Thiruvananthapuram respectively of the total inequality. The positive contribution of these factors contributed to pro-rich inequality in BG testing, while age (− 12% in Kasaragod, − 14% in Alappuzha), religion (− 18% in Kasaragod, − 22% in Alappuzha), and caste (− 14% in Alappuzha) contributed to pro-poor inequality.

## Discussion

We found a range in testing for BP and BG by health personnel in the previous year ranging from 77% for BG of the population surveyed in Alappuzha, as compared to 92% for BP in Kasaragod. Overall, there was a positive socioeconomic gradient in BP and BG testing in three of the four districts with testing coverage being concentrated among wealthier populations. This is concerning in light of the global evidence that suggests that NCD risk factors cluster in population with low socioeconomic status in LMICs [20] our own study found that less educated groups had greater self-reported prevalence of high BP and BG [32]. Research using a large national dataset found that undiagnosed hypertension was more common among poorer, less educated (male) individuals even when they visited a health facility [50]. A study focused on Kerala found greater prevalence of raised BP among men as compared to women [11]. There is clearly a need to match coverage of testing to burden in Kerala, as elsewhere in the country, particularly given that testing is associated with significantly better awareness and treatment (of BP) [51]. Such research should be mindful of likely variations in prevalence by age, location, and other factors [52].

Decomposition analysis revealed that socioeconomic inequality in BP testing was largely attributable to wealth and education and (with greater contributions to testing inequality in districts where coverage was lower). It was noteworthy that differences by sex and religion were not seen at the district level. While our previous work has found greater testing among women [32], at the district level, efforts may be more successful at identifying those left behind by placing attention on those with lower education levels or those who are caste minorities. This becomes more salient in light of the newly announced Aardram 2.0 program in the state which redoubles efforts to operationalize the national NCD primary care package [13]. This will entail population enumeration of all individuals aged 30 and over, administration of a Community Based Assessment Checklist (CBAC) and motivation for BP and BG screening (as well as cancer screening). A recent cost effectiveness study (using data from neighbouring Tamil Nadu and Punjab in northern India) concluded that if 70% of diabetes and hypertension cases could be treated at the Health and Wellness primary health centres, population based screening of the type attempted in Aardram could be deemed cost effective [53]. Another modelling study at the national level has concluded that for BG, false positive rates would be high, and exclusion of poor and rural populations are likely [54]. In order to achieve 70% and higher treatment coverage levels and simultaneously address equity gaps, the program may benefit from placing emphasis on populations with lower education and those of lower economic status. As such population subgroups often tend to sequester in terms of living and working environments, campaigns must be strengthened to identify and engage these populations. Moreover, Kerala’s decentralisation reforms have also been associated with a number of local level initiatives that may have had an impact on health service utilisation in general and on NCD care-seeking in particular. It will be important to understand the role of these efforts.

Other dimensions of inequality (such as religion or sex) did not contribute significantly to socioeconomic inequality in BP and BG testing. More than reflecting testing, this perhaps pertains to the relationship between these other variables and socioeconomic status in general (i.e., the recursively and strength of relationship between wealth (inequality) and caste as well as wealth (inequality) and education may be greater than that between wealth (inequality) and sex or religion per se). Research in northern India has found that males were being excluded from population-based screening efforts, hypothesized to be attributed to the emphasis of health systems on maternal and child care in those regions [38]. Given that NCD control programs have been longer running in India’s southern states like Kerala, the lack of sex and religion-based exclusion warrants further study and may offer insights for programming in other parts of the country.

## Limitations

The study has a number of important findings, however there are various limitations to the study that should be addressed. First, as it is common in studies on care utilization, researcher relied on self-reported data in defining the use of both testing and treatment. This could potentially bias the findings, especially if there were cases of misreporting [22]. Additionally, during the survey, participants sometimes responded on behalf of other family members who were not present at the time. As a result, the results may have been underestimated. Second, because the data in this study is cross-sectional in nature, it is difficult to discern temporal patterns in inequality and inequality in the usage of screening or testing. It’s also worth noting that the factors underlying reported disparities in NCDs testing are not necessarily causative. Third, we acknowledge there could be external factors that might impact the testing ability of individuals, for example, accessing testing facilities could depend on availability of good roads and public transportation, and working hours of the facility. Another limitation of this study is that there may be additional variables that are uniquely relevant in each district context. In the interest of comparison across districts, we have not included them. District focussed research should be carried out to shed further light on this.

## Conclusions

While the magnitude of socioeconomic inequality in NCD risk factor testing did not appear to be very high in four Kerala districts, the levels were, however, statistically significant in three of them. Further exploration is now needed on the public health significance of these inequalities, the relative contributions of education, wealth contribute and family history to NCD risk factors, as well as the role of local outreach projects. These analyses might be helpful in insuring testing access is truly universal and responds to the distribution of NCD burden.

## Supplementary Information

**Additional file 1: Table A1.** BP and BG measured by doctor/nurse/other medical personnel in the previous year in Kerala. **Table A2.** Inequality ratio and Inequality gap of proportion of respondents screened for blood pressure and blood glucose in the districts of Kerala. **Table A3.** Decomposition of CCI in BP testing in Kasaragod and Alappuzha. **Table A4.** Decomposition of CCI in BP testing in Kollam and Thiruvananthapuram. **Table A5.** Decomposition of CCI in BG testing in Kasaragod and Alappuzha. **Table A6.** Decomposition of CCI in BG testing in Kollam and Thiruvananthapuram.

## Data Availability

All datasets used for supporting the conclusions of this paper are available from the corresponding author on request.

## Abbreviations

NCDs: Non-Communicable Diseases
BP: Blood Pressure
BG: Blood Glucose
CCI: Concentration Index
HBP: High Blood Pressure
HBG: High Blood Glucose
CVD: Cardiovascular diseases
NPCDCS: National Programme for Prevention and Control of Cancer, Diabetes, CVD and Stroke
SDGs: Sustainable Development Goals
FHC: Family Health Centre
PHC: Primary Health Centre
PSU: Primary Sampling Unit
NFHS: National Family Health Survey
NHM: National Health Mission
PCA: Principal component analysis
SES: Socioeconomic status
