# Supplementary material for: Decomposing socioeconomic inequality in blood pressure and blood glucose testing: evidence from four districts in Kerala, India
Source: Int J Equity Health. 2022 Sep 9;21:128. doi: 10.1186/s12939-022-01737-x (PMC9461212; doi:10.1186/s12939-022-01737-x)
Supplement: Supplementary file 1 — Additional file 1: Table A1. BP and BG measured by doctor/nurse/other medical personnel in the previous year in Kerala. Table A2. Inequality ratio and Inequality gap of proportion of respondents screened for blood pressure and blood glucose in the districts of Kerala. Table A3. Decomposition of CCI in BP testing in Kasaragod and Alappuzha. Table A4. Decomposition of CCI in BP testing in Kollam and Thiruvananthapuram. Table A5. Decomposition of CCI in BG testing in Kasaragod and Alappuzha. Table A6. Decomposition of CCI in BG testing in Kollam and Thiruvananthapuram. [file 12939_2022_1737_MOESM1_ESM.docx]

**Supplementary Tables**

**Appendix Tables and Figures**

| **Table A1: BP and BG measured by doctor/nurse/other medical personnel in the previous year in Kerala** | | | | | |
| --- | --- | --- | --- | --- | --- |
| Districts | Blood pressure testing | | Blood glucose testing | | No. of respondents (N) |
|  | % | 95% Conf. Interval | % | 95% Conf. Interval |  |
| Kasaragod | 91.75 | [90.11,93.13] | 88.76 | [86.59,90.61] | 1631 |
| Alappuzha | 82.47 | [80.50,84.28] | 77.36 | [74.20,80.24] | 1607 |
| Kollam | 84.98 | [81.76,87.71] | 81.36 | [77.66,84.56] | 1594 |
| Thiruvananthapuram | 85.61 | [83.18,87.74] | 83.24 | [80.96,85.30] | 1535 |
| Total | 85.86 | [84.42,87.19] | 82.53 | [80.95,84.00] | 6367 |

| **Table A2: Inequality ratio and Inequality gap of proportion of respondents screened for blood pressure and blood glucose in the districts of Kerala** | | | | | | | | | | |
| --- | --- | --- | --- | --- | --- | --- | --- | --- | --- | --- |
|  | Blood pressure testing | | | | | | Blood glucose testing | | | |
|  | | Poorest quintile | Richest quintile | Inequality ratio (rich/poor) | Inequality gap  (rich-poor) | | Poorest quintile | Richest quintile | Inequality ratio (rich/poor) | Inequality gap  (rich-poor) |
| Kasaragod | | 89.8 | 92.6 | 1.03 | | 2.80 | 84.63 | 89.65 | 1.06 | 5.02 |
| Alappuzha | | 80.3 | 86.9 | 1.08 | | 6.54 | 75.98 | 82.25 | 1.08 | 6.27 |
| Kollam | | 81.8 | 90.0 | 1.10 | | 8.20 | 76.61 | 88.82 | 1.16 | 12.22 |
| Thiruvananthapuram | | 81.0 | 92.3 | 1.14 | | 11.36 | 77.41 | 91.34 | 1.18 | 13.93 |

Table A2 presents the wealth inequality ratio (that is the relative difference in the proportion of respondents measured for blood pressure and blood glucose between extreme wealth quintile), and the inequality gap (i. e., the absolute difference in the proportion of respondents measured for blood pressure between extreme wealth quintile) in the districts of Kerala. The result of rich-poor ratio and rich-poor difference revealed that respondents from the richest households were more likely to get tested for blood pressure and blood glucose than the respondents from the poorest households in all the selected districts of Kerala. More particularly, the value of rich-poor ratio and rich-poor difference for the blood pressure testing was highest in Thiruvananthapuram (1.14 and 11.36), followed by Kollam (1.10 and 8.20), and Alappuzha (1.08 and 6.54). Similarly, the value of rich-poor ratio and rich-poor difference for the blood glucose testing was highest in Thiruvananthapuram (1.18 and 13.93), followed by Kollam (1.16 and 12.22), Alappuzha (1.08 and 6.27). This means that socio-economic inequality in blood pressure and blood glucose testing was higher among affluent class of household in the selected districts of Kerala.

| **Table A3: Decomposition of CCI in BP testing in Kasaragod and Alappuzha** | | | | | | | | |
| --- | --- | --- | --- | --- | --- | --- | --- | --- |
|  | Kasaragod | | | | Alappuzha | | | |
|  | Elasticity | CI | Absolute | % Contribution | Elasticity | CI | Absolute | % Contribution |
| **Age** |  |  |  |  |  |  |  |  |
| 30-44 |  |  |  |  |  |  |  |  |
| 45-59 | 0.1009 | 0.0090 | 0.0009 | 3.5 | 0.1476 | -0.0146 | -0.0021 | -2.6 |
| 60+ | 0.2307 | -0.0375 | -0.0087 | -33.1 | 0.3425 | -0.0233 | -0.0080 | -9.6 |
| **Sex** |  |  |  |  |  |  |  |  |
| Male |  |  |  |  |  |  |  |  |
| Female | 0.1417 | -0.0093 | -0.0013 | -5.1 | 0.1903 | -0.0001 | 0.0000 | 0.0 |
| **Education** |  |  |  |  |  |  |  |  |
| Illiterate |  |  |  |  |  |  |  |  |
| Primary | 0.0064 | -0.0559 | -0.0004 | -1.4 | -0.0036 | -0.0089 | 0.0000 | 0.0 |
| Secondary | 0.0947 | -0.2224 | -0.0211 | -80.6 | -0.0404 | -0.2674 | 0.0108 | 13.0 |
| Higher secondary | 0.0974 | 0.3196 | 0.0311 | 119.2 | -0.0096 | 0.2954 | -0.0028 | -3.4 |
| **Wealth quintile** |  |  |  |  |  |  |  |  |
| Poorest |  |  |  |  |  |  |  |  |
| Poorer | 0.0057 | -0.295 | -0.0017 | -6.4 | -0.0174 | -0.3529 | 0.0061 | 7.4 |
| Middle | 0.0149 | -0.0518 | -0.0008 | -3.0 | 0.0394 | -0.1766 | -0.007 | -8.4 |
| Richer | 0.0191 | 0.2795 | 0.0053 | 20.5 | 0.0487 | 0.2489 | 0.0121 | 14.6 |
| Richest | 0.0106 | 0.7057 | 0.0075 | 28.8 | 0.0777 | 0.7507 | 0.0584 | 70.4 |
| **Religion** |  |  |  |  |  |  |  |  |
| Hindu | -0.0071 | -0.1054 | 0.0008 | 2.9 | 0.0179 | -0.1907 | -0.0034 | -4.1 |
| Non-Hindu |  |  |  |  |  |  |  |  |
| **Caste** |  |  |  |  |  |  |  |  |
| SC/ST |  |  |  |  |  |  |  |  |
| OBC | 0.0301 | -0.071 | -0.0021 | -8.2 | 0.1297 | -0.1203 | -0.0156 | -18.8 |
| Others | 0.033 | 0.1384 | 0.0046 | 17.5 | 0.0359 | 0.1823 | 0.0066 | 7.9 |
| **Family History of NCDs** |  |  |  |  |  |  |  |  |
| No |  |  |  |  |  |  |  |  |
| Yes | 0.0587 | 0.1307 | 0.0077 | 29.4 | 0.0624 | 0.1468 | 0.0092 | 11.0 |
| Total CI |  |  | 0.0218 |  |  |  | 0.0643 |  |
| Actual CI |  |  | 0.0261 |  |  |  | 0.0829 |  |
| Residual |  |  | 0.0043 | 16.5 |  |  | 0.0186 | 22.4 |

| **Table A4: Decomposition of CCI in BP testing in Kollam and Thiruvananthapuram** | | | | | | | | |
| --- | --- | --- | --- | --- | --- | --- | --- | --- |
|  | Kollam | | | | Thiruvananthapuram | | | |
|  | Elasticity | CI | Absolute | % Contribution | Elasticity | CI | Absolute | % Contribution |
| **Age** |  |  |  |  |  |  |  |  |
| 30-44 |  |  |  |  |  |  |  |  |
| 45-59 | 0.1495 | -0.0166 | -0.0025 | -3.2 | 0.1076 | 0.0292 | 0.0031 | 3.6 |
| 60+ | 0.2859 | -0.0012 | -0.0003 | -0.4 | 0.2000 | 0.0265 | 0.0053 | 6.1 |
| **Sex** |  |  |  |  |  |  |  |  |
| Male |  |  |  |  |  |  |  |  |
| Female | 0.2740 | 0.0067 | 0.0018 | 2.4 | 0.2067 | -0.0146 | -0.0030 | -3.5 |
| **Education** |  |  |  |  |  |  |  |  |
| Illiterate |  |  |  |  |  |  |  |  |
| Primary | 0.0019 | -0.0041 | 0.0000 | 0.0 | -0.0039 | -0.0076 | 0.0000 | 0.0 |
| Secondary | 0.0412 | -0.2459 | -0.0101 | -13.2 | 0.0835 | -0.1862 | -0.0155 | -17.9 |
| Higher secondary | 0.0756 | 0.3133 | 0.0237 | 30.9 | 0.1444 | 0.3167 | 0.0457 | 52.7 |
| **Wealth quintile** |  |  |  |  |  |  |  |  |
| Poorest |  |  |  |  |  |  |  |  |
| Poorer | -0.0066 | -0.4128 | 0.0027 | 3.6 | 0.0038 | -0.3152 | -0.0012 | -1.4 |
| Middle | 0.0302 | -0.0536 | -0.0016 | -2.1 | 0.0243 | 0.0406 | 0.0010 | 1.1 |
| Richer | 0.0548 | 0.3375 | 0.0185 | 24.2 | 0.0046 | 0.3193 | 0.0015 | 1.7 |
| Richest | 0.0607 | 0.6460 | 0.0392 | 51.2 | 0.0671 | 0.6149 | 0.0413 | 47.5 |
| **Religion** |  |  |  |  |  |  |  |  |
| Hindu | -0.0563 | -0.1328 | 0.0075 | 9.8 | -0.0176 | 0.0546 | -0.0010 | -1.1 |
| Non-Hindu |  |  |  |  |  |  |  |  |
| **Caste** |  |  |  |  |  |  |  |  |
| SC/ST |  |  |  |  |  |  |  |  |
| OBC | -0.0160 | -0.0255 | 0.0004 | 0.5 | -0.0129 | -0.0574 | 0.0007 | 0.9 |
| Others | -0.0413 | 0.1263 | -0.0052 | -6.8 | 0.0138 | 0.2305 | 0.0032 | 3.7 |
| **Family History of NCDs** |  |  |  |  |  |  |  |  |
| No |  |  |  |  |  |  |  |  |
| Yes | 0.1363 | 0.0877 | 0.0120 | 15.6 | 0.1511 | 0.1556 | 0.0235 | 27.1 |
| Total CI |  |  | 0.0861 |  |  |  | 0.1046 |  |
| Actual CI |  |  | 0.0766 |  |  |  | 0.0868 |  |
| Residual |  |  | -0.0095 | -12.4 |  |  | -0.0178 | -20.5 |

| **Table A5: Decomposition of CCI in BG testing in Kasaragod and Alappuzha** | | | | | | | | |
| --- | --- | --- | --- | --- | --- | --- | --- | --- |
|  | Kasaragod | | | | Alappuzha | | | |
|  | Elasticity | CI | Absolute | % Contribution | Elasticity | CI | Absolute | % Contribution |
| **Age** |  |  |  |  |  |  |  |  |
| 30-44 |  |  |  |  |  |  |  |  |
| 45-59 | 0.1135 | 0.0090 | 0.0010 | 1.8 | 0.1441 | -0.0146 | -0.0021 | -2.9 |
| 60+ | 0.2093 | -0.0375 | -0.0078 | -13.9 | 0.3656 | -0.0233 | -0.0085 | -11.6 |
| **Sex** |  |  |  |  |  |  |  |  |
| Male |  |  |  |  |  |  |  |  |
| Female | 0.0850 | -0.0093 | -0.0008 | -1.4 | 0.1518 | -0.0001 | 0.0000 | 0.0 |
| **Education** | |  |  |  |  |  |  |  |
| Illiterate |  |  |  |  |  |  |  |  |
| Primary | 0.0084 | -0.0559 | -0.0005 | -0.8 | 0.0021 | -0.0089 | 0.0000 | 0.0 |
| Secondary | -0.0053 | -0.2224 | 0.0012 | 2.1 | 0.0483 | -0.2674 | -0.0129 | -17.6 |
| Higher secondary | -0.0047 | 0.3196 | -0.0015 | -2.7 | 0.1055 | 0.2954 | 0.0312 | 42.4 |
| **Wealth quintile** | |  |  |  |  |  |  |  |
| Poorest |  |  |  |  |  |  |  |  |
| Poorer | 0.0184 | -0.2950 | -0.0054 | -9.6 | -0.0177 | -0.3529 | 0.0062 | 8.5 |
| Middle | 0.0262 | -0.0518 | -0.0014 | -2.4 | 0.0263 | -0.1766 | -0.0046 | -6.3 |
| Richer | 0.0756 | 0.2795 | 0.0211 | 37.4 | 0.0331 | 0.2489 | 0.0082 | 11.2 |
| Richest | 0.0363 | 0.7057 | 0.0256 | 45.4 | 0.0707 | 0.7507 | 0.0531 | 72.2 |
| **Religion** |  |  |  |  |  |  |  |  |
| Hindu | 0.0974 | -0.1054 | -0.0103 | -18.2 | 0.0854 | -0.1907 | -0.0163 | -22.2 |
| Non-Hindu | |  |  |  |  |  |  |  |
| **Caste** |  |  |  |  |  |  |  |  |
| SC/ST |  |  |  |  |  |  |  |  |
| OBC | 0.0539 | -0.0710 | -0.0038 | -6.8 | 0.2797 | -0.1203 | -0.0336 | -45.8 |
| Others | 0.0395 | 0.1384 | 0.0055 | 9.7 | 0.1275 | 0.1823 | 0.0232 | 31.7 |
| **Family History of NCDs** | | |  |  |  |  |  |  |
| No |  |  |  |  |  |  |  |  |
| Yes | 0.0737 | 0.1307 | 0.0096 | 17.0 | 0.0780 | 0.1468 | 0.0115 | 15.6 |
| Total CI |  |  | 0.0325 |  |  |  | 0.0554 |  |
| Actual CI |  |  | 0.0565 |  |  |  | 0.0734 |  |
| Residual |  |  | 0.0240 | 42.5 |  |  | 0.0180 | 24.6 |

| **Table A6: Decomposition of CCI in BG testing in Kollam and Thiruvananthapuram** | | | | | | | | |
| --- | --- | --- | --- | --- | --- | --- | --- | --- |
|  | Kollam | | | | Thiruvananthapuram | | | |
|  | Elasticity | CI | Absolute | % Contribution | Elasticity | CI | Absolute | % Contribution |
| **Age** |  |  |  |  |  |  |  |  |
| 30-44 |  |  |  |  |  |  |  |  |
| 45-59 | 0.1948 | -0.0166 | -0.0032 | -3.6 | 0.1089 | 0.0292 | 0.0032 | 2.9 |
| 60+ | 0.3437 | -0.0012 | -0.0004 | -0.4 | 0.2113 | 0.0265 | 0.0056 | 5.1 |
| **Sex** |  |  |  |  |  |  |  |  |
| Male |  |  |  |  |  |  |  |  |
| Female | 0.2318 | 0.0067 | 0.0016 | 1.7 | 0.1861 | -0.0146 | -0.0027 | -2.5 |
| **Education** | |  |  |  |  |  |  |  |
| Illiterate |  |  |  |  |  |  |  |  |
| Primary | 0.0028 | -0.0041 | 0.0000 | 0.0 | -0.0043 | -0.0076 | 0.0000 | 0.0 |
| Secondary | -0.0004 | -0.2459 | 0.0001 | 0.1 | 0.0794 | -0.1862 | -0.0148 | -13.4 |
| Higher secondary | 0.0917 | 0.3133 | 0.0287 | 31.8 | 0.0567 | 0.3167 | 0.0179 | 16.3 |
| **Wealth quintile** | |  |  |  |  |  |  |  |
| Poorest |  |  |  |  |  |  |  |  |
| Poorer | 0.0224 | -0.4128 | -0.0093 | -10.3 | -0.0002 | -0.3152 | 0.0000 | 0.0 |
| Middle | 0.0257 | -0.0536 | -0.0014 | -1.5 | 0.0525 | 0.0406 | 0.0021 | 1.9 |
| Richer | 0.0475 | 0.3375 | 0.0160 | 17.8 | 0.0247 | 0.3193 | 0.0079 | 7.2 |
| Richest | 0.0854 | 0.6460 | 0.0552 | 61.1 | 0.0960 | 0.6149 | 0.0590 | 53.7 |
| **Religion** |  |  |  |  |  |  |  |  |
| Hindu | -0.0598 | -0.1328 | 0.0079 | 8.8 | -0.0100 | 0.0546 | -0.0005 | -0.5 |
| Non-Hindu | |  |  |  |  |  |  |  |
| **Caste** |  |  |  |  |  |  |  |  |
| SC/ST |  |  |  |  |  |  |  |  |
| OBC | 0.0134 | -0.0255 | -0.0003 | -0.4 | 0.0166 | -0.0574 | -0.0010 | -0.9 |
| Others | -0.0020 | 0.1263 | -0.0002 | -0.3 | 0.0208 | 0.2305 | 0.0048 | 4.4 |
| **Family History of NCDs** | | |  |  |  |  |  |  |
| No |  |  |  |  |  |  |  |  |
| Yes | 0.1669 | 0.0877 | 0.0146 | 16.2 | 0.1181 | 0.1556 | 0.0184 | 16.7 |
| Total CI |  |  | 0.1093 |  |  |  | 0.0999 |  |
| Actual CI |  |  | 0.0903 |  |  |  | 0.1100 |  |
| Residual |  |  | -0.0190 | -21.0 |  |  | 0.01009 | 9.2 |
